# From imaging to clinical outcome: dual-region CT radiomics predicting FOXM1 expression and prognosis in hepatocellular carcinoma

**DOI:** 10.3389/fonc.2023.1278467

**Published:** 2023-09-28

**Authors:** Xianyu Chen, Yongsheng Tang, Donghao Wu, Ruixi Li, Zhiqun Lin, Xuhui Zhou, Hezhen Wang, Hang Zhai, Junming Xu, Xianjie Shi, Guangquan Zhang

**Affiliations:** ^1^ Department of Hepatobiliary and Pancreatic Surgery, The Eighth Affiliated Hospital of Sun Yat-sen University, Shenzhen, China; ^2^ Department of Hepatic Surgery, Liver Transplantation Center, The Third Affiliated Hospital of Sun Yat-sen University, Guangzhou, China; ^3^ Department of Medical Oncology, The Third Affiliated Hospital of Sun Yat-sen University, Guangzhou, China; ^4^ Department of Radiology, The Eighth Affiliated Hospital of Sun Yat-sen University, Shenzhen, China; ^5^ Department of Radiology, The Second Affiliated Hospital of Zhengzhou University, Zhengzhou, China

**Keywords:** HCC, FOXM1, radiomics, prognosis, immune microenvironment

## Abstract

**Background:**

Liver cancer, especially hepatocellular carcinoma (HCC), remains a significant global health challenge. Traditional prognostic indicators for HCC often fall short in providing comprehensive insights for individualized treatment. The integration of genomics and radiomics offers a promising avenue for enhancing the precision of HCC diagnosis and prognosis.

**Methods:**

From the Cancer Genome Atlas (TCGA) database, we categorized mRNA of HCC patients by Forkhead Box M1 (FOXM1) expression and performed univariate and multivariate studies to pinpoint autonomous HCC risk factors. We deployed subgroup, correlation, and interaction analyses to probe FOXM1’s link with clinicopathological elements. The connection between FOXM1 and immune cells was evaluated using the CIBERSORTx database. The functions of FOXM1 were investigated through analyses of Gene Ontology (GO) and the Kyoto Encyclopedia of Genes and Genomes (KEGG). After filtering through TCGA and the Cancer Imaging Archive (TCIA) database, we employed dual-region computed tomography (CT) radiomics technology to noninvasively predict the mRNA expression of FOXM1 in HCC tissues. Radiomic features were extracted from both tumoral and peritumoral regions, and a radiomics score (RS) was derived. The performance and robustness of the constructed models were evaluated using 10-fold cross-validation. A radiomics nomogram was developed by incorporating RS and clinical variables from the TCGA database. The models’ discriminative abilities were assessed using metrics such as the area under the curve (AUC) of the receiver operating characteristic curves (ROC) and precision-recall (PR) curves.

**Results:**

Our findings emphasized the overexpression of FOXM1 as a determinant of poor prognosis in HCC and illustrated its impact on immune cell infiltration. After selecting arterial phase CT, we chose 7 whole-tumor features and 3 features covering both the tumor and its surroundings to create WT and WP models for FOXM1 prediction. The WT model showed strong predictive capabilities for FOXM1 expression by PR curve. Conversely, the WP model did not demonstrate the good predictive ability. In our study, the radiomics score (RS) was derived from whole-tumor regions on CT images. The RS was significantly associated with FOXM1 expression, with an AUC of 0.918 in the training cohort and 0.837 in the validation cohort. Furthermore, the RS was correlated with oxidative stress genes and was integrated with clinical variables to develop a nomogram, which demonstrated good calibration and discrimination in predicting 12-, 36-, and 60-month survival probabilities. Additionally, bioinformatics analysis revealed FOXM1’s potential role in shaping the immune microenvironment, with its expression linked to immune cell infiltration.

**Conclusion:**

This study highlights the potential of integrating FOXM1 expression and radiomics in understanding HCC’s complexity. Our approach offers a new perspective in utilizing radiomics for non-invasive tumor characterization and suggests its potential in providing insights into molecular profiles. Further research is needed to validate these findings and explore their clinical implications in HCC management.

## 1 Introduction

Liver cancer stands as a formidable global health challenge, being the sixth most prevalent tumor and the third leading cause of cancer-related mortality worldwide (1). Hepatocellular carcinoma (HCC), which constitutes 75%-85% of primary liver cancer cases, is particularly concerning (2). Despite the availability of diverse treatment modalities for HCC, including hepatic resection, liver transplantation, ablation, targeted therapy, and combination therapies, postoperative recurrence remains a daunting obstacle, largely attributed to HCC’s intricate pathological underpinnings (3). Traditional prognostic markers for HCC encompass clinicopathological traits, diagnostic laboratory markers like alpha-fetoprotein (AFP), and imaging techniques such as computed tomography (CT), magnetic resonance imaging (MRI), and ultrasound (4, 5). However, these markers often fall short in guiding individualized precision treatment. Consequently, there’s a pressing clinical need to unearth novel prognostic indicators, leveraging cutting-edge technologies like genomics, proteomics, and radiomics.

Genetic research plays a pivotal role in deciphering the architecture and functionality of living organisms and has been widely utilized across diverse medical domains. It has been applied in diverse clinical areas, encompassing clinical diagnostics, pharmaceutical advancement, and disease prediction (6–9). The Forkhead Box M1 (FOXM1) gene, pivotal in orchestrating cell cycle gene expression, is indispensable for DNA replication and mitosis (10). It’s intricately involved in cell proliferation, DNA damage repair, checkpoint responses, liver regeneration, and oxidative stress management (11–13). Analyses of gene expression profiles in HCC patients have pinpointed an overexpression of FOXM1 as a harbinger of poor HCC prognosis (14). Gadolinium ethoxybenzyl diethylenetriamine pentaacetic acid (Gd-EOB-DTPA) serves as a liver-specific MRI contrast agent, and its hepatic uptake offers insights into the functional status of hepatocytes. According to research, decreased uptake of Gd-EOB-DTPA and elevated serum AFP levels have been linked to activation of the oncogene FOXM1 and poorer prognosis (15). Therefore, FOXM1 has emerged as a pivotal gene garnering our attention.

Radiomics, a burgeoning non-invasive diagnostic modality, facilitates dynamic tumor monitoring by mining high-dimensional, quantitative metric features from medical images (16–18). Its potential shines especially bright in oncology, given the unique hemodynamic and metabolic signatures tumors exhibit (19). Recent studies underscore the diagnostic and predictive prowess of radiomics, especially when analyzing both tumoral and peritumoral regions in HCC, offering insights into the tumor microenvironment (18, 20, 21). Moreover, radiomics holds promise in pre-surgically forecasting gene expression, a capability that could revolutionize preoperative prognostic evaluations and therapeutic decision-making in cancer patients (22).

In light of the above, our study pioneers a novel strategy to non-invasively forecast FOXM1 mRNA expression in HCC tissues via dual-region CT radiomics. We aim to discern the relationship between our radiomics model and patient prognosis, and harness bioinformatics to shed light on the molecular mechanisms tethering FOXM1 expression to the immune microenvironment. A PubMed search with the keywords ‘radiomics’, ‘liver cancer’ or ‘hepatocellular carcinoma’, and ‘FOXM1’ yielded no pertinent studies, underscoring the groundbreaking nature of our research endeavor.

## 2 Methods

### 2.1 Data acquisition and preprocessing

**Figure 1:**
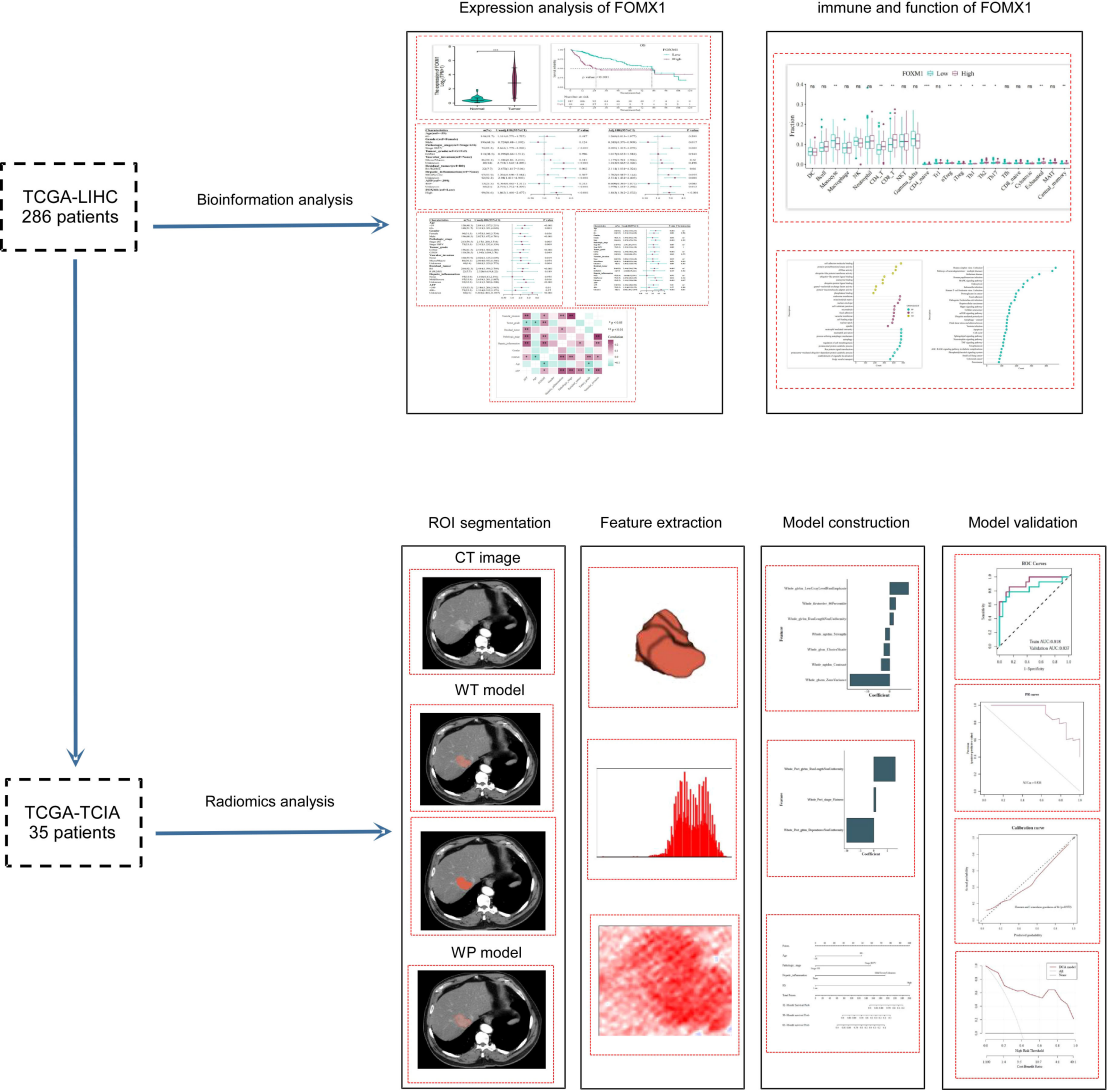
Study Design and Workflow. The flowchart outlined the research process, starting with the initial cohort of 286 TCGA-LIHC patients subjected to bioinformatics analysis. An intersection with the TCIA database yielded a subset of 35 patients, who were then analyzed using radiomics techniques.

The study’s workflow is depicted in **Figure 1**. Initially, a cohort of 377 HCC patients was sourced from the Cancer Genome Atlas Liver Hepatocellular Carcinoma (TCGA-LIHC) dataset (https://portal.gdc.cancer.gov/). To ensure the reliability and relevance of the data, we applied several exclusion criteria:

(1) Absence of survival status information (n = 1).
(2) Unavailability of overall survival (OS) time or an OS time of less than 30 days (n = 28).
(3) Missing crucial variables such as stage, grade, and tumor residue status (n = 41).
(4) Primary medical diagnosis not being HCC (n = 13).

After applying these criteria, the cohort was narrowed down to 294 patients. Out of these, 286 patients with overlapping clinical and genetic data in the TCGA-LIHC database were earmarked for bioinformatics analysis.

For the radiomics analysis, 75 sets of medical imaging data were obtained from the Cancer Imaging Archive (TCIA: https://www.cancerimagingarchive.net/). The primary selection criterion was the availability of arterial phase enhanced CT data. Patients with either poor-quality images or postoperative images were excluded (n = 34). After cross-referencing with the TCGA-LIHC dataset, a total of 35 patients were retained for the radiomics analysis. To ensure consistency in the radiomic analysis, the selected CT images underwent normalization to achieve uniform intensity values. Regions of interest (ROIs) were delineated to encompass both the whole-tumor and the peritumoral regions. Experienced radiologists reviewed the segmented images to ensure accuracy.

### 2.2 FOXM1 expression analysis

To investigate the differential mRNA expression levels of FOXM1 between tumor tissues and their adjacent counterparts, we sourced the gene expression data in the HTSeq-FPKM (Fragments Per Kilobase of transcript per Million mapped reads) format from the TCGA-LIHC dataset. This data was then converted from HTSeq-FPKM format to TPM (transcripts per million reads) format to ensure a more accurate representation of gene expression. Following this, a log2 transformation was applied to the TPM data to stabilize the variance and make the data more amenable to statistical analysis. Utilizing the “ggplot2” package in R, we visually represented the disparities in FOXM1 expression between tumor and adjacent tissues, aiming to elucidate the potential role of FOXM1 in hepatocellular carcinoma progression.

### 2.3 Survival analysis

To discern the influence of FOXM1 expression on the OS outcomes of HCC patients, we employed the Kaplan–Meier method. The log-rank test was applied to determine the statistical significance of the differences in survival curves. Both of these analyses were conducted using the “survival” package in R. The median survival time, representing the time point at which 50% of the patient population remains alive, was computed, and the corresponding point survival rates were derived. To provide a comprehensive and visually interpretable representation of these results, we utilized the “survminer” package in R, which facilitated the summarization and visualization of the survival analysis outcomes.

### 2.4 Univariate and multivariate Cox regression analysis

To elucidate the relationship between FOXM1 expression, clinicopathological factors, and survival outcomes in HCC patients, both univariate and multivariate Cox regression analyses were conducted. In the univariate analysis, each clinicopathological factor, including age, gender, pathologic stage, tumor grade, vascular invasion, residual tumor status, hepatic inflammation, AFP levels, along with FOXM1 expression levels, was individually assessed for its association with survival outcomes. Following the univariate analysis, variables that demonstrated a p-value less than 0.05 were incorporated into the multivariate Cox regression analysis to determine their independent prognostic significance. In this context, a hazard ratio (HR) value greater than 1 indicated that the variable was a risk factor for poor survival, while an HR value less than 1 suggested its role as a protective factor. All statistical analyses related to the Cox regression were executed using the “survival” package in R, and the results were visualized using the “forestplot” package.

### 2.5 Subgroup stratification and interaction analysis

To delve deeper into the nuanced effects of FOXM1 on patient prognosis across various clinical subgroups, an exploratory subgroup analysis was undertaken. This stratification aimed to discern whether the prognostic significance of FOXM1 expression varied across different patient categories, such as those defined by age, gender, pathologic stage, tumor grade, vascular invasion, and other pertinent clinicopathological factors. The univariate Cox regression model was employed for each subgroup to evaluate the association between FOXM1 expression and survival outcomes. Furthermore, to ascertain potential interactions between FOXM1 and other covariates, a likelihood ratio test was conducted. This interaction analysis provided insights into whether the effect of FOXM1 on prognosis was modified by the presence of other variables. All statistical computations for the subgroup and interaction analyses were executed using the “cmprsk”, “survival”, and “forestplot” packages in R.

### 2.6 Immune cell infiltration analysis in relation to FOXM1 expression

To gain a comprehensive understanding of the tumor microenvironment in relation to FOXM1 expression, we employed the CIBERSORTx database (https://cibersortx.stanford.edu/). This tool facilitated the estimation of the abundance of various immune cell types within the tumor samples based on their gene expression profiles. Specifically, we assessed the infiltration levels of a myriad of immune cells, encompassing B cells, CD4+ T cells, naive CD4+ T cells CD8+ T cells, naive CD8 cells, Tfh cells, Th1 cells, Th2 cells, Th17 cells, Tregs, Tr1 cells, exhausted T cells, cytotoxic T cells, macrophages, monocytes, NKT cells, NK cells, neutrophils, MAIT cells, central memory T cells, effector memory T cells and dendritic cells. Subsequently, we stratified the samples into high and low FOXM1 expression groups to compare the immune cell infiltration levels between them. This comparison was executed using the “limma” package in R. For statistical significance, notations “***”, “**”, and “*” were used to represent p-values of<0.001,<0.01, and <0.05, respectively.

### 2.7 Differential gene expression and enrichment analysis

To elucidate the biological implications of FOXM1 expression in hepatocellular carcinoma, we embarked on a comprehensive functional annotation of genes associated with differential FOXM1 expression. Leveraging the Gene Ontology (GO) analysis, we were able to categorize these genes based on their functional attributes, spanning across three main domains: biological process (BP), molecular function (MF), and cellular component (CC). This categorization aids in deciphering the broader biological context and significance of these genes. Furthermore, to gain insights into the potential pathways influenced by FOXM1, we turned to the Kyoto Encyclopedia of Genes and Genomes (KEGG). For our study, patients were stratified based on their FOXM1 expression levels, and a subsequent functional enrichment analysis was conducted. Utilizing the R package “clusterProfiler”, we identified the top 10 significantly enriched pathways for each of the GO domain (BP, CC, and MF) and the top 30 for KEGG. Any GO term or KEGG pathway with a q-value less than 0.05 was deemed statistically significant.

### 2.8 Radiomic feature extraction and analysis

**Figure 2:**
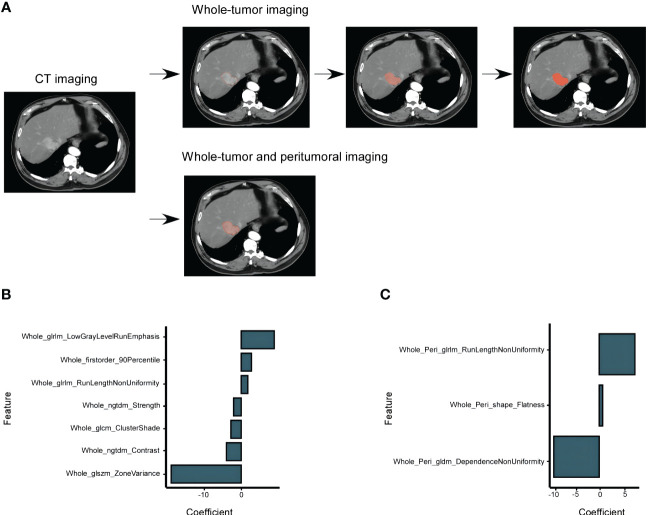
HCC CT Imaging Selection and Models Construction Based on the TCIA-LIHC Databases. **(A)** Selection of enhanced CT images in the arterial phase, showcasing the whole-tumor and peritumoral Regions of Interest (ROIs). The peritumoral ROIs were derived from a 3 mm area beyond the lesion. **(B)** Determination of FOXM1 expression using the minimum AIC rule, based on 7 imaging characteristics from the whole-tumor group. **(C)** FOXM1 expression determination using 3 imaging characteristics from both the whole-tumor and peritumoral groups.

For the radiomic analysis, ROIs were meticulously delineated around the tumor lesions of 35 patients using the ITK-SNAP software (3.6.0 version). This task was undertaken by two seasoned radiologists, and they operated under a double-blind protocol to ensure unbiased results. For a comprehensive feature extraction, both whole-tumor (WT) radiomics features and whole-tumor and peritumoral (WP) radiomics features were extracted. The latter involved outlining an additional 3-mm peritumoral area surrounding the tumor (**Figure 2A**). All images were recorded in the Digital Medical Imaging and Communication (DICOM) format. Prior to feature extraction, a crucial pre-processing step was executed to harmonize the intensity levels across all CT images, ensuring consistency. Subsequently, the DICOM images were fed into the A.K. software (Artificial Intelligence Kit, Version 3.3.0, GE Life Science, Institute of Precision Medicine) to extract the radiomics features.

To ensure the reproducibility and reliability of the extracted features, a Z-score normalization was applied: (z) = (x - μ)/σ, where x is the given value, μ is the mean, and σ is the standard deviation. This method aids in standardizing the intensity values of the features. The R “caret” package was employed to standardize the datasets from TCIA and TCGA.

### 2.9 Feature selection and predictive modeling

To predict FOXM1 expression using radiomics features, we employed the maximum relevance minimum redundancy (mRMR) algorithm. This method identifies pivotal features by assessing their correlation with FOXM1 and inter-feature correlations. This rigorous process ensured that only the most pertinent and reproducible radiomics features were retained for subsequent analyses. Post preliminary screening, a subset of significant radiomics features was selected. Using these, a logistic regression model was constructed, with feature selection further refined based on the Akaike Information Criterion (AIC). Two models were derived: WT Model from selected whole-tumor features and WP Model from both whole-tumor and peritumoral features. Inter-observer agreement of the extracted features was gauged using inter-class correlation coefficients (ICCs). An ICC value ≥ 0.75 indicated good agreement, 0.51 to 0.74 signified moderate agreement, and<0.50 represented poor agreement. The R packages “mRMRe” and “stats” facilitated feature selection and model construction and R packages “irr”.

### 2.10 Radiomics score derivation and nomogram development

To delve deeper into the prognostic potential of radiomics, we derived a radiomics score (RS) from the probability values predicted by the WT model. A threshold of 0.550 was set as the cutoff to categorize patients. Spearman correlation analysis was employed to discern the relationship between RS and oxidative stress genes. We spotlighted the top 50 genes based on the absolute value of their correlation coefficients, ensuring they also had a p-value below 0.05. To enhance the prognostic precision, a nomogram was crafted. This was achieved by amalgamating the RS with pertinent clinical variables from the TCGA dataset, namely age, pathologic stage, and hepatic inflammation. Leveraging the Cox regression model, we formulated nomogram that could predict survival probabilities at 12, 36, and 60 months. For computational purposes, the R package “survminer” facilitated the derivation of the RS. The “stats” package aided in analyzing the association with oxidative stress genes, while the “survival” package was instrumental in the survival analysis related to RS.

### 2.11 Model performance and validation

The performance of the WT model, WP model, and the nomogram was assessed across both training and validation datasets using 10-fold cross-validation to minimize overfitting and enhance the models’ generalizability. We evaluated the models using key metrics such as accuracy (ACC), specificity (SPE), sensitivity (SEN), positive predictive value (PPV), and negative predictive value (NPV). Furthermore, the discriminative abilities of the models were determined by computing the area under the curve (AUC) of the receiver operating characteristic (ROC) and precision-recall (PR) curves. The Brier score, which quantifies the accuracy of probabilistic predictions, was also determined for each model. To validate the calibration of the models, we applied the Hosmer-Lemeshow goodness-of-fit test, assessing the agreement between the predicted probabilities of high FOXM1 expression and the observed outcomes. The clinical utility of the models was further evaluated using decision curve analysis (DCA), which quantified the net benefits at various threshold probabilities. For computational procedures, we utilized the R “pROC” package for ROC analysis and the “rms” package for calibration curve plotting and the Hosmer-Lemeshow test. The decision curve analysis was executed using the “dca.R” function.

### 2.12 Statistical analysis

To discern differences in FOXM1 expression between tumor and adjacent tissues, the Wilcoxon rank sum test was employed. The association between FOXM1 expression and clinical pathological characteristics was probed using the Wilcoxon signed-rank test, Kruskal-Wallis test, and logistic regression. The relationship between FOXM1 and both RS and oxidative stress genes was assessed via Spearman correlation analysis. The Kaplan-Meier method was utilized to determine the prognostic significance of FOXM1 expression and the constructed models. The Cohen kappa coefficient was employed to gauge the interobserver agreement in the assessment of imaging features. AUC values were furnished with 95% confidence intervals (CIs) and juxtaposed using Delong’s test. All statistical evaluations were executed using SPSS (version 28, IBM, Armonk, NY, USA) and R software (version 4.3.0). For model evaluation, R packages “pROC”, “measures”, “ResourceSelection”, and “modEvA” were harnessed, while the “Irr” package facilitated the computation of the ICC. A two-sided p-value less than 0.05 was deemed statistically significant.

## 3 Results

### 3.1 Baseline characteristics and FOXM1 expression in HCC patients

**Figure 3:**
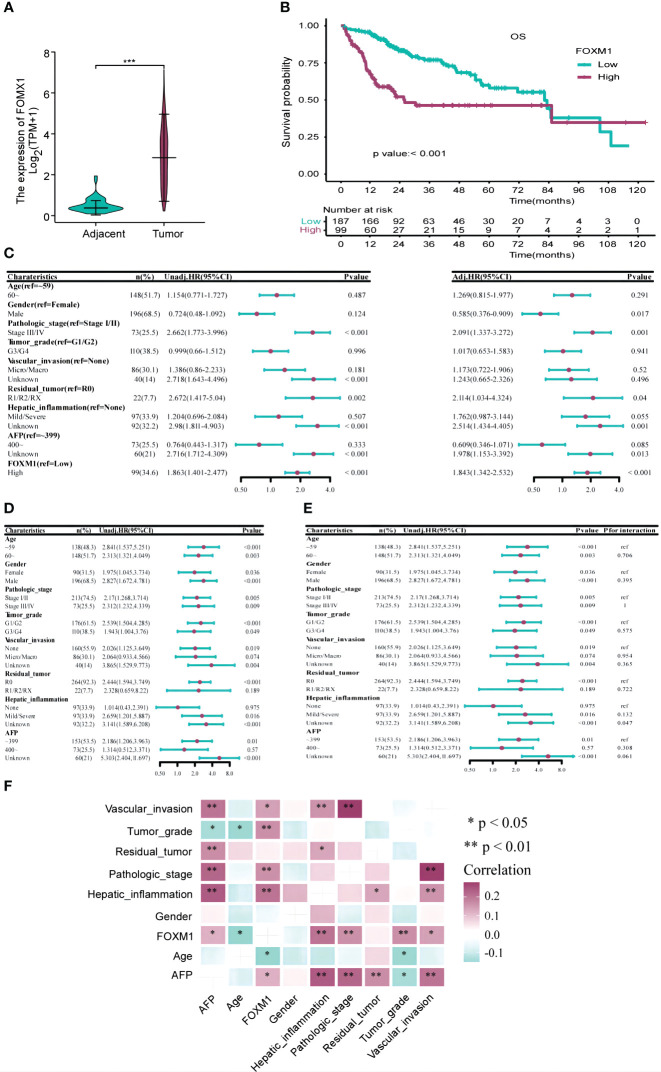
The Correlation Between FOXM1 and Clinicopathological Characteristics and Prognosis in the TCGA-LIHC Databases. **(A)** Elevated FOXM1 expression in HCC compared to adjacent tissues. **(B)** Kaplan-Meier curve illustrating the association between high FOXM1 expression and poor prognosis. **(C)** Univariate and multivariate Cox analyses identifying risk factors for prognosis. **(D)** Univariate Cox analysis assessing the impact of FOXM1 expression levels on prognosis across different clinicopathological subgroups. **(E)** Cox analysis evaluating the influence of clinicopathological characteristics on the relationship between FOXM1 expression and overall survival (OS). **(F)** Correlation matrix showcasing the relationship between FOXM1 expression and various clinicopathological characteristics. Red indicates a positive correlation, while light green indicates a negative correlation.

To evaluate the differential expression of FOXM1 between HCC and adjacent non-tumorous tissues, we sourced FOXM1 mRNA levels from the TCGA database. Our analysis revealed a pronounced upregulation of FOXM1 mRNA in tumor samples compared to their non-tumorous counterparts (P< 0.001) (**Figure 3A**).

We extracted clinicopathological data of LIHC patients from the TCGA database, encompassing variables such as age, gender, tumor stage, grade, vascular invasion, residual tumor status, hepatic inflammation, and AFP levels. The distribution of these clinical characteristics across low and high FOXM1 expression groups is detailed in **Table 1**. Notably, FOXM1 expression exhibited significant associations with age (P = 0.016), tumor stage (P = 0.009), grade (P = 0.008), vascular invasion (P = 0.012), and hepatic inflammation (P = 0.001). However, gender, residual tumor status, and AFP levels did not show any significant variance between the FOXM1 expression groups (P > 0.05). Kaplan-Meier survival curves underscored the adverse prognostic implications of elevated FOXM1 expression, with the high-expression group manifesting a notably diminished survival trajectory (**Figure 3B**). Specifically, the median survival duration was markedly shorter in the high FOXM1 expression cohort (25.6 months) compared to their low-expression counterparts (81.9 months).

**Table 1:** Comparing the clinicopathological characteristics of HCC patients in different FOXM1 expression groups.

Variables	Total (n = 286)	Low (n = 187)	High (n = 99)	p
Age, n (%)				0.016
~59	138 (48)	80 (43)	58 (59)	
60~	148 (52)	107 (57)	41 (41)	
Gender, n (%)				0.37
Female	90 (31)	55 (29)	35 (35)	
Male	196 (69)	132 (71)	64 (65)	
Pathologic_stage, n (%)				0.009
Stage I/II	213 (74)	149 (80)	64 (65)	
Stage III/IV	73 (26)	38 (20)	35 (35)	
Tumor_grade, n (%)				0.008
G1/G2	176 (62)	126 (67)	50 (51)	
G3/G4	110 (38)	61 (33)	49 (49)	
Vascular_invasion, n (%)				0.012
None	160 (56)	112 (60)	48 (48)	
Micro/Macro	86 (30)	57 (30)	29 (29)	
Unknown	40 (14)	18 (10)	22 (22)	
Residual_tumor, n (%)				0.68
R0	264 (92)	174 (93)	90 (91)	
R1/R2/RX	22 (8)	13 (7)	9 (9)	
Hepatic_inflammation, n(%)				0.001
None	97 (34)	73 (39)	24 (24)	
Mild/Severe	97 (34)	67 (36)	30 (30)	
Unknown	92 (32)	47 (25)	45 (45)	
AFP, n (%)				0.117
~399	153 (53)	107 (57)	46 (46)	
400~	73 (26)	47 (25)	26 (26)	

Univariate Cox regression pinpointed FOXM1 as a potent risk determinant for overall survival (OS) with an HR of 1.863 (95% CI: 1.401-2.477, p<0.001). Other clinical parameters, including tumor stage and residual tumor presence, also emerged as significant predictors of OS. Subsequent multivariate analysis reaffirmed the independent prognostic relevance of FOXM1 (HR=1.843, 95%CI: 1.342-2.532, p<0.001), tumor stage, and residual tumor status (**Figure 3C**). Subgroup evaluations revealed that high FOXM1 expression consistently portended poorer OS across various clinical stratum, including age, gender, pathological stage, and tumor grade (**Figure 3D**). Interaction assessments further corroborated that these factors did not modulate the relationship between FOXM1 expression and patient survival outcomes (**Figure 3E**). Subsequently, a correlation analysis was performed to investigate the associations between FOXM1 expression and various clinicopathological factors in HCC. FOXM1 expression demonstrated significant correlations with age, tumor stage, tumor grade, vascular invasion, hepatic inflammation, and AFP levels (**Figure 3F**).

Further correlation analyses were conducted to elucidate the relationships between FOXM1 expression and a spectrum of clinicopathological attributes in HCC. Significant correlations emerged between FOXM1 expression and variables such as age, pathologic stage, tumor grade, vascular invasion, hepatic inflammation, and AFP levels. In summation, our findings robustly establish FOXM1 as a pivotal molecular marker, intricately linked with the adverse clinical trajectory of HCC patients.

### 3.2 Immunological implications and functional roles of FOXM1 in HCC

**Figure 4:**
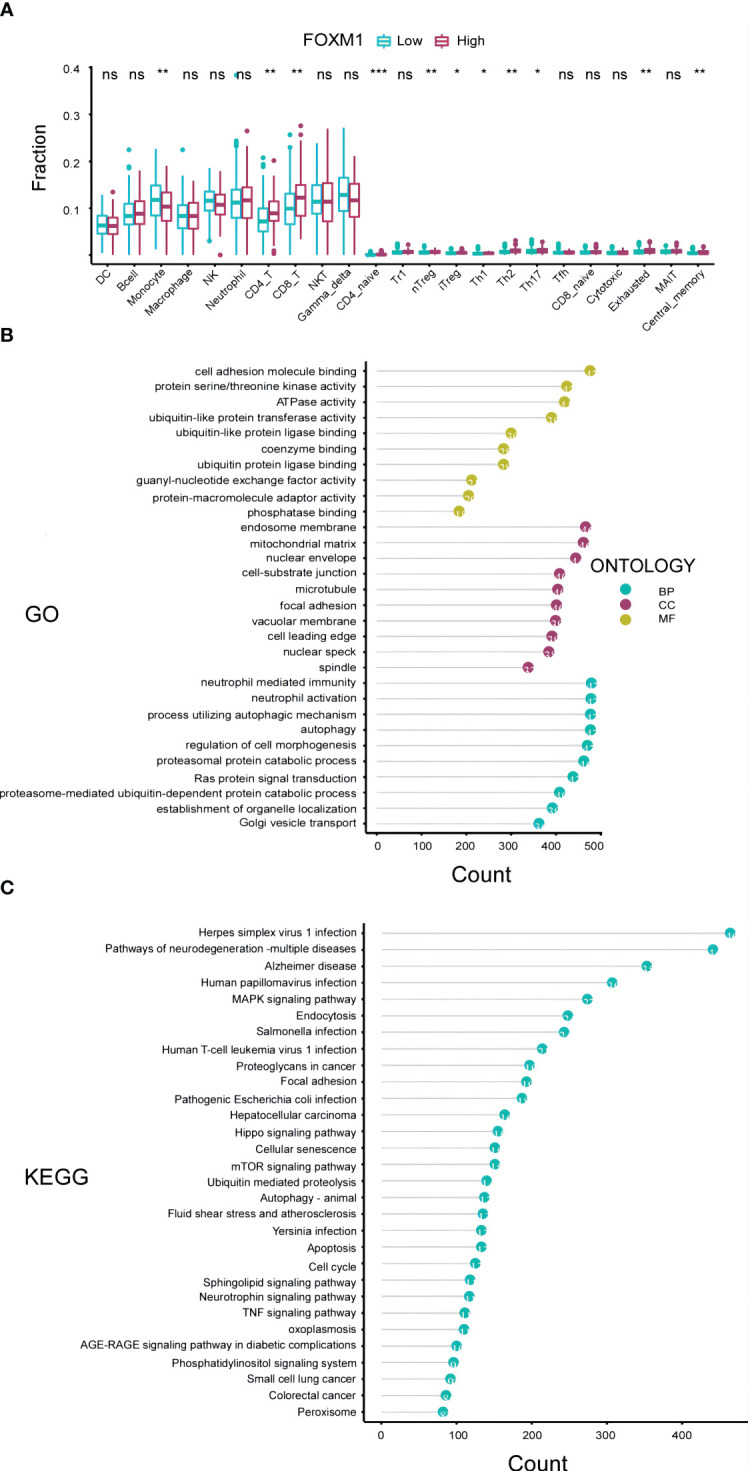
Immunological and Functional Analysis of FOXM1 in the TCGA-LIHC Cohort. **(A)** Box plot representation of the variations in 24 immune cells among patients with different FOXM1 expression levels, as determined by the Immune Cell Abundance Identifier. Notations: ns indicates no significance; *P< 0.05; **P< 0.01; ***P< 0.001. **(B)** Top 10 results from the Gene Ontology (GO) analysis based on differentially expressed genes associated with FOXM1, categorized into Biological Process (BP), Cell Component (CC), and Molecular Function (MF). **(C)** Top 30 results from the Kyoto Encyclopedia of Genes and Genomes (KEGG) analysis based on FOXM1 differentially expressed genes.

Changes in FOXM1 expression can influence the functionality of the immune system, potentially driving the onset and progression of liver cancer. To delve into the interplay between FOXM1 expression and immunity in HCC, we utilized the Immune Cell Abundance Identifier (ImmuCellAI) algorithms and the CIBERSORTx database to assess the relative proportions of various immune cells in relation to FOXM1 expression levels. Our findings highlighted a pronounced increase in monocyte cell infiltration and a marked decrease in exhausted cells in the cohort with diminished FOXM1 expression (P<0.01) (**Figure 4A**). This suggests that elevated FOXM1 expression provides an immunosuppressive microenvironment for HCC. Notably, the effector memory T cells were not included in **Figure 4A** due to their negligible expression levels.

A profound understanding of gene functions is pivotal for deciphering the tumor immunological microenvironment and the intricate processes governing tumor cell growth, invasion, and metastasis. To further elucidate the biological significance of the FOXM1 gene in HCC, we embarked on a functional enrichment analysis, encompassing both GO and KEGG enrichment, based on TCGA data (**Figures 4B, C**). Our GO analysis, visualizing the top 10 significantly enriched pathways, revealed that the differentially expressed genes (DEGs) in the FOXM1 high expression groups were predominantly associated with molecular functions such as cell adhesion molecule binding, protein serine kinase activity, and ATPase activity. Concurrently, the KEGG analysis spotlighted the DEGs’ inclination towards pathways like the MAPK signaling pathway, endocytosis, and other pivotal signaling cascades. In essence, the combined GO and KEGG analyses underscored the potential of FOXM1 in modulating cellular adhesion processes and metabolic activities. This sheds light on novel avenues for research into tumor cell migration and the intricate tumor microenvironment.

### 3.3 Radiomics analysis: from image selection to model evaluation and comparison

Utilizing the combined resources of the TCIA and TCGA databases, we embarked on a comprehensive analysis to discern specific radiological features that could predict FOXM1 expression status. From the entirety of the ROIs, we extracted a pool of 107 imaging features. The mRMR algorithm, renowned for its capability to consider both the relevance between features and the predicted variable and the redundancy between features, was employed for feature selection. This process distilled the features down to two distinct models: the WT model and the WP model.

In line with the AIC criteria, the WT model retained 7 pivotal features, whereas the WP model harnessed 3 features. **Figures 2B, C**, through a histogram, graphically showcased the coefficients of these rigorously selected features. The radiomics signatures, formulated from these features, can be articulated mathematically as:

WT model:


$$\begin{array}{l} {\text{RS}}_{\text{WT}}=3.030603145-1.943492788\times \text{Whole}\_\text{ngtdm}\_\text{Strength}-17.91038915\\ \times \text{Whole}\_\text{glszm}\_\text{ZoneVariance}-3.789451111\\ \times \text{Whole}\_\text{ngtdm}\_\text{Contrast}+8.449892409\\ \times \text{Whole}\_\text{glrlm}\_\text{LowGrayLevelRunEmphasis}-2.642860539\\ \times \text{Whole}\_\text{glcm}\_\text{ClusterShade}+1.683785937\\ \times \text{Whole}\_\text{glrlm}\_\text{RunLengthNonUniformity}+2.627375082\\ \times \text{Whole}\_\text{firstorder}\_90\text{Percentile} \end{array}$$

WP Model:


$$\begin{array}{l} {\text{RS}}_{\text{WP}}=-1.730305966-10.06574986\\ \times \text{Whole}\_\text{Peri}\_\text{gldm}\_\text{DependenceNonUniformity}+7.905895664\\ \times \text{Whole}\_\text{Peri}\_\text{glrlm}\_\text{RunLengthNonUniformity}+0.765734038\\ \times \text{Whole}\_\text{Peri}\_\text{shape}\_\text{Flatness} \end{array}$$

The performance of the radiomics models was rigorously evaluated in both training and validation sets using a 10-fold cross-validation approach. The metrics employed for this evaluation encompassed ACC, SPE, SEN, PPV, and NPV, as detailed in **Table 2**.

**Table 2:** Estimating the effectiveness of the prediction models.

Model		AUC [95%CI]	ACC	SPE	SEN	PPV	NPV
WT model	Train set	0.918 [0.828-1]	0.857	0.857	0.857	0.8	0.9
	Testing set	0.837 [0.681-0.992]	0.829	0.786	0.857	0.786	0.857
WP model	Train set	0.803 [0.654-0.951]	0.8	1	0.667	0.667	1
	Testing set	0.789 [0.637-0.942]	0.771	0.929	0.667	0.65	0.933

WT model, whole-tumor model; WP model, whole-tumor and peritumoral model; ACC, accuracy; SPE, specificity; SEN, sensitivity; PPV, positive predictive value; NPV, negative predictive value.

**Figure 5:**
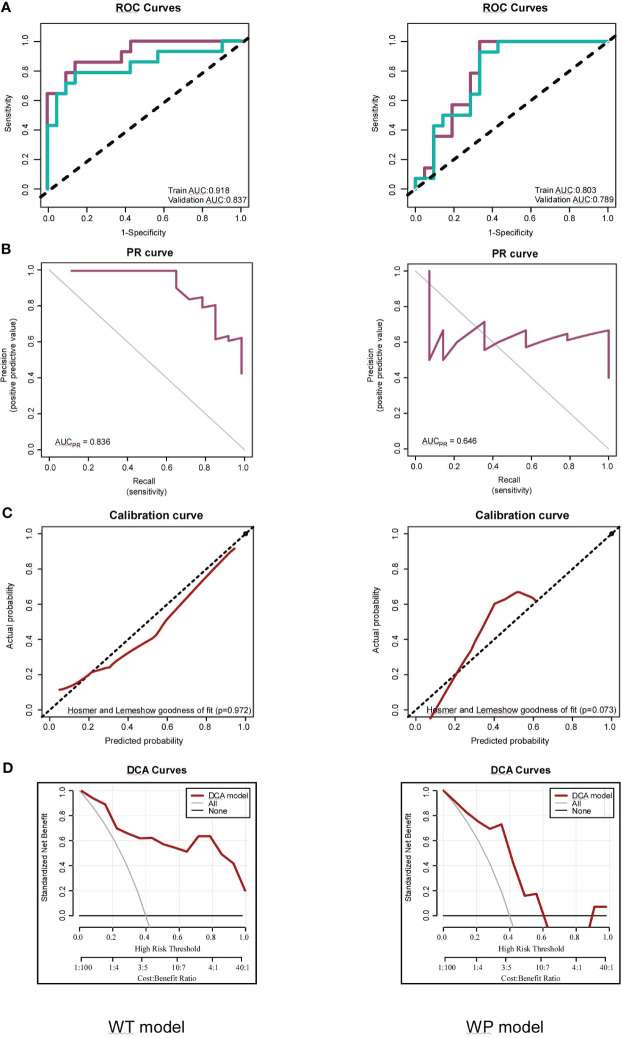
Assessments of the Whole-Tumor (WT) and Whole-Tumor and Peritumoral (WP) Models for FOXM1 Expression Prediction. **(A)** Receiver Operating Characteristic (ROC) curve illustrating the predictive performance of the WT and WP models in both Training and Testing sets. **(B)** Precision-Recall (PR) curve highlighting the WT and WP models’ precision in predicting FOXM1 expression. **(C)** Calibration plot comparing the predicted probability of FOXM1 expression to the actual observed values in the WT and WP models. **(D)** Decision Curve Analysis (DCA) evaluating the clinical applicability of the WT and WP models in forecasting FOXM1 expression.

In the WT model, we observed commendable performance across both the training and testing datasets for all metrics. Conversely, the WP model showcased favorable performance only in ACC, SPE, and NPV. Notably, the two radiomics models demonstrated impressive predictive performance with Brier scores of 0.111 (WT model) and 0.184 (WP model) in the training sets. The WT model achieved high AUC values in both the training (AUC = 0.918) and testing sets (AUC = 0.837). In comparison, the WP model exhibited slightly lower AUC values in the training (AUC = 0.841) and testing sets (AUC = 0.821), as visualized in **Figure 5A**. Intriguingly, the Delong test revealed no significant difference between the ROC curves of the WT and WP models. The AUC values of the PR curve for the WT and WP models stood at 0.836 and 0.646, respectively, as depicted in **Figure 5B**, reinforcing the superior predictive value of the WT model.

The calibration curves, illustrated in **Figure 5C**, underscored the strong consistency between the predicted probability of high FOXM1 expression and the actual observed values for both WT and WP models, as validated by the Hosmer-Lemeshow goodness-of-fit test across training and validation cohorts. The clinical utility of these models was further emphasized through a decision curve analysis conducted for each model in their respective training datasets (**Figure 5D**). As the high-risk threshold range expanded, both radiomics models exhibited an uptick in the standard net benefit, translating to enhanced clinical effectiveness.

To ensure the robustness and reproducibility of our radiomics features, a consistency evaluation was conducted. A subset of 10 samples was randomly selected and delineated by an alternate radiologist. The ICC was employed to assess the consistency of the radiomics features extracted from the ROIs delineated by the two radiologists. In our study, all the selected radiomics features showcased an ICC value of ≥ 0.75, underscoring the high reliability and reproducibility of our feature extraction process. This consistency assessment ensures that the radiomics features utilized in our models are not only predictive but also reproducible across different delineations, enhancing the clinical applicability of our findings. These results are shown in the **Table 3**.

**Table 3:** Investigating the ICC values of radiomic features in both the WT and WP models.

Radiomics feature	ICC value
Whole_ngtdm_Strength	0.779457303
Whole_glszm_ZoneVariance	0.998512609
Whole_ngtdm_Contrast	0.986828133
Whole_glrlm_LowGrayLevelRunEmphasis	0.970547479
Whole_glcm_ClusterShade	0.958116068
Whole_glrlm_RunLengthNonUniformity	0.995672962
Whole_firstorder_90Percentile	0.999657409
Whole_Peri_gldm_DependenceNonUniformity	0.996035776
Whole_Peri_glrlm_RunLengthNonUniformity	0.996697895
Whole_Peri_shape_Flatness	0.91228024

WT model, whole-tumor model; WP model, whole-tumor and peritumoral model.

In summation, our radiomics analysis, rooted in a robust methodology and comprehensive feature selection, offers promising models for predicting FOXM1 expression in liver cancer. The high ICC values further bolster the reliability of these models, paving the way for their potential clinical applications.

### 3.4 Prognostic nomogram and clinical implications

In our pursuit to further delineate the prognostic implications of radiomics in HCC, we focused on RS, derived from the probability values predicted by the WT model. Employing a cutoff value of 0.550, we stratified our cohort into high-score and low-score groups.

**Figure 6:**
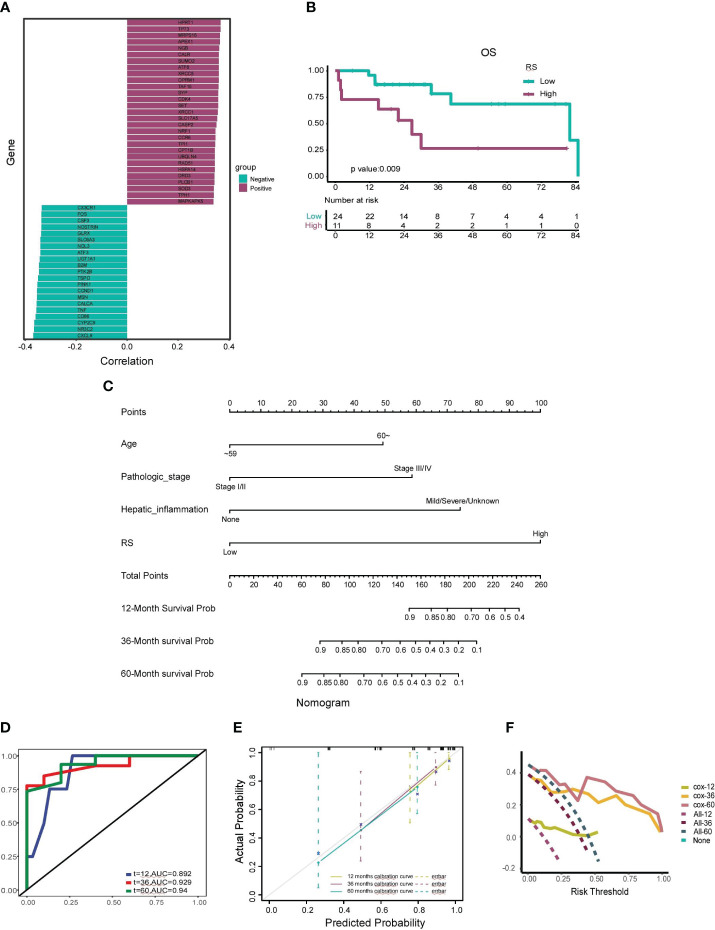
Construction and Validation of the Prognostic Nomogram. **(A)** Correlation analysis between the radiomics score (RS) and the top 25 positively correlated and 25 negatively correlated oxidative stress genes. Genes with positive correlations are depicted in dark green, and genes with negative correlations are shown in fuchsia. **(B)** Kaplan-Meier curve illustrating the association between a high-RS group and shorter median survival time, as well as a worse survival rate. **(C)** Nomogram developed to estimate the probability of survival for HCC patients at 12, 36, and 60 months, incorporating factors such as age, pathologic stage, hepatic inflammation, and RS. **(D)** Receiver Operating Characteristic (ROC) curve assessing the nomogram’s predictive capability for patient prognosis and overall survival (OS) at 12, 36, and 60 months. **(E)** Calibration curves adjusting for discrepancies between the nomogram’s predicted and actual survival rates at 12, 36, and 60 months. **(F)** Decision Curve Analysis (DCA) evaluating the clinical utility of the nomogram for predicting patient survival at 12, 36, and 60 months.

Recognizing the profound influence of oxidative stress in amplifying the production of reactive oxygen species, which culminates in cellular damage, we incorporated oxidative stress genes into our investigative framework. Furthermore, extant literature has underscored a nexus between FOXM1 and oxidative stress (23). Given FOXM1’s pivotal role as a quintessential transcription factor in myriad cancers, its interplay with oxidative stress potentially elucidates a mechanistic pathway through which FOXM1 might modulate tumor progression. This premise steered us to probe the relationship between RS and the expression profiles of oxidative stress genes. Intriguingly, we discerned a marked positive correlation between RS and genes such as CCNA2, MAPK7 and CDK1. Conversely, genes like CSF1, IL18 and ABCB1 exhibited a significant negative correlation with RS. The top 25 genes with positive and negative correlation respectively, delineated by their absolute correlation coefficient, are showcased in **Figure 6A**.

The prognostic prowess of RS was further appraised using Kaplan-Meier curves. The low-score group manifested a median survival time of 65.61 months, whereas the high-score group clocked a median survival time of 34.16 months. As illustrated in **Figure 6B**, the high-score group bore a conspicuously diminished OS rate in juxtaposition with the low-score group (p=0.009).

To augment the clinical applicability of our insights, we crafted a radiomics nomogram to prognosticate the 12-, 36-, and 60-month OS rates of HCC patients. This nomogram amalgamated RS with salient clinicopathological determinants, encompassing age, pathologic stage, and hepatic inflammation. The coefficients of these variables are portrayed in **Figure 6C**.

The veracity of our nomogram was scrutinized using ROC curves, which unveiled AUC values of 0.892, 0.929, and 0.94 for the 12-, 36-, and 60-month forecasts, respectively (**Figure 6D**). Calibration curves further endorsed the fidelity of our nomogram, evincing a robust concordance between the prognosticated and actual 12-, 36-, and 60-month OS rates (**Figure 6E**). The decision curve analysis, encapsulated in **Figure 6F**, accentuated the clinical utility of our nomogram, underscoring substantial net benefits across diverse threshold probabilities at 12, 36, and 60 months. The validation cohort was harnessed to fortify the resilience of our model, safeguarding against potential overfitting and corroborating its precision, calibration, and clinical pertinence.

## 4 Discussion

In this comprehensive study, we embarked on an innovative exploration into the realm of hepatocellular carcinoma (HCC), focusing on the potential prognostic implications of FOXM1 gene expression and its non-invasive prediction using dual-region CT radiomics technology. Our findings underscored the significant differential expression of FOXM1 between tumor and adjacent tissues, reinforcing its potential role in HCC pathogenesis. Furthermore, the RS, derived from the probability values predicted by the WT model, provided a novel avenue for stratifying patients based on their FOXM1 expression levels. Notably, the RS demonstrated a significant correlation with oxidative stress genes, shedding light on the intricate molecular interplay underpinning HCC progression.

The integration of radiomics and genomics in our study unveiled a promising approach for individualized precision treatment in HCC. By harnessing the power of advanced imaging and bioinformatics tools, we were able to bridge the gap between macroscopic imaging features and microscopic genetic alterations, offering a holistic perspective on HCC’s complex landscape.

The role of FOXM1 in various cancers has been a focal point in oncological research. Liu et al. found that FOXM1 promotes cell proliferation in hepatocellular carcinoma, gastric cancer, and colorectal cancer by upregulating STMN1, emphasizing its significance in cell proliferation and tumor genesis (24). Similarly, Zhang et al. further elucidated the inhibitory potential of miR-370 on acute myeloid leukemia progression via FOXM1 targeting (25). In the context of pancreatic cancer, FOXM1 has been implicated in stem cell renewal and proliferation, further promoting pancreatic intraepithelial neoplasia, as reviewed by Quan et al. (26). Furthermore, in glioblastoma, FOXM1 has been shown to promote tumorigenesis by mediating the nuclear translocation of β-catenin in the absence of conventional Wnt/β-catenin pathway activation (27).

Zooming into HCC, Kopanja et al. spotlighted FOXM1’s instrumental role in Ras-driven HCC progression, particularly emphasizing its role in the survival of HCC cells with stem cell characteristics via regulation of reactive oxygen species (28). Xia et al. identified FOXM1 expression as a pivotal independent determinant affecting postoperative recurrence and survival in HCC patients. Their insights into the mechanism revealed FOXM1’s role in promoting liver cancer cell invasion and metastasis by upregulating MMP-7, RhoC, and ROCK1, with HBx further amplifying FOXM1 expression through the ERK/CREB pathway (29). Weiler et al. provided insights into the synergistic action of YAP and FOXM1, leading to chromosomal instability in HCC (30). Hu et al. expanded on this intricate relationship, suggesting a mechanism where interferon-a inhibits HIF1a signaling in liver cancer cells by suppressing FosB transcription. This established a high glucose microenvironment promoting CD27 transcription in T cells via the mTOR-FOXM1 signaling pathway, thereby amplifying the therapeutic efficacy of PD-1 on HCC (31). Our findings, which accentuate FOXM1’s overexpression as a harbinger of poor prognosis in HCC, align with these studies, further solidifying FOXM1’s detrimental contribution to HCC progression.

The influence of FOXM1 on oxidative stress has been documented, with studies suggesting that FOXM1 can regulate oxidative stress and is involved in the expression of antioxidant genes. This role has been observed across six cancers, including pancreatic cancer, colorectal cancer, head and neck squamous cell carcinoma, lung cancer, prostate cancer, and breast cancer, promoting their progression (32). Our research adds a new dimension to this narrative in HCC, suggesting FOXM1’s potential modulation of oxidative stress levels through genes like CCNA2, MAPK7 and CDK1.

Radiomics has emerged as a powerful tool in oncology, offering insights into tumor characteristics and potential therapeutic targets. Song et al. demonstrated the added diagnostic value of combining imaging parameters with clinical features in identifying the presence of a micropapillary component in lung adenocarcinomas (33). Li et al. constructed a predictive model using preoperative T2-weighted MRI images, RNA-seq, and clinical data from 652 glioma patients across three independent cohorts. Their model reliably predicted patient survival times and aided in the preoperative assessment of macrophage infiltration in brain gliomas (34).

In the specific context of HCC radiomics, Feng et al. constructed a model comprising 11 radiomic features from preoperative liver-enhanced CT results of 365 adult HCC patients from three medical centers. Their model showed promising predictive capabilities for the macrotrabecular-massive subtype of HCC (35). Xia et al. developed a radiomics model using preoperative registration or subtraction CT images, demonstrating significant value in predicting microvascular invasion in HCC patients (36). Our study innovatively bridges radiomic features with FOXM1 gene expression, aiming to predict HCC patients FOXM1 expression and OS from preoperative enhanced CT images.

In essence, while our study aligns with several established findings in the literature, it also introduces novel insights, particularly in the integration of radiomics and molecular profiling in HCC. The comprehensive approach adopted in our research, combining FOXM1 expression, oxidative stress, and radiomics, sets a precedent for future endeavors in this direction.

The integration of radiomics with molecular profiling, as demonstrated in our study, holds transformative potential for the clinical management of HCC. By non-invasively predicting FOXM1 mRNA expression in HCC tissues using dual-region CT radiomics technology, clinicians can gain invaluable insights into the tumor’s molecular landscape even before surgical intervention. This predictive capability can guide therapeutic decisions, allowing for more personalized treatment strategies tailored to the individual patient’s molecular profile. Moreover, the correlation we established between FOXM1 expression and prognosis can serve as a valuable prognostic marker. By identifying patients with high FOXM1 expression, clinicians can anticipate a potentially aggressive tumor behavior and adjust treatment regimens accordingly. This could lead to more aggressive therapeutic interventions for high-risk patients, while sparing low-risk patients from unnecessary treatments. Furthermore, our findings on the association between FOXM1 expression and the immune microenvironment can have profound implications for immunotherapy. As FOXM1 potentially shapes the tumor immune landscape, understanding its expression can provide insights into a patient’s likely response to immune checkpoint inhibitors and other immunotherapeutic agents. This could pave the way for more effective combination therapies, where FOXM1-targeted treatments are combined with immunotherapies to enhance therapeutic outcomes.

Our study, while offering valuable insights into the role of FOXM1 in HCC, has certain limitations. The primary constraint is the sample size, as our research on radiomics and genomics was predominantly based on retrospective data sourced from the TCGA and TCIA databases. This retrospective nature necessitates the validation of our findings in larger, multi-center cohorts to ensure broader applicability. Additionally, our imaging-based predictive nomogram model did not incorporate certain clinical parameters and biochemical markers, such as surgical approaches, albumin, and transaminases. This omission might have restricted our analysis’s depth, potentially overlooking intricate relationships between these factors and patient outcomes. Furthermore, while we delved into the influence of FOXM1 on prognosis and immunity, our study did not explore the specific mechanisms underpinning its effects in detail. The absence of experimental validation further underscores this limitation.

Given these limitations, future research should prioritize expanding the patient cohort, incorporating a wider range of clinical and biochemical parameters, and delving deeper into the mechanistic pathways of FOXM1 in HCC. Experimental studies could provide a more granular understanding and validation of our findings. In conclusion, our study, despite its constraints, sheds light on the potential of integrating radiomics with molecular profiling in HCC, paving the way for more personalized and effective therapeutic strategies.

## Data availability statement

The original contributions presented in the study are publicly available. This data can be found here: https://osf.io/43dpx/?view_only=b206df4680654678a6bdcfa4ca092fbb.

## Ethics statement

The manuscript presents research on animals that do not require ethical approval for their study. Written informed consent was obtained from the individual(s) for the publication of any potentially identifiable images or data included in this article.

## Author contributions

XC: Writing – original draft, Data curation, Formal Analysis. YT: Data curation, Writing – original draft, Formal Analysis. DW: Data curation, Writing – original draft, Formal Analysis. RL: Writing – original draft, Formal Analysis. ZL: Writing – original draft, Formal Analysis. XZ: Writing – review & editing, Conceptualization, Software. HW: Writing – original draft, Conceptualization, Software. HZ: Writing – original draft, Formal Analysis. JX: Writing – original draft, Formal Analysis. XS: Writing – review & editing, Funding acquisition, Methodology, Project administration, Resources. GZ: Writing – review & editing, Funding acquisition, Methodology, Project administration, Resources.

## Glossary

**Table:**

HCC	Hepatocellular carcinoma
FOXM1	Forkhead Box M1
Gd-EOB-DTPA	Gadolinium ethoxybenzyl diethylenetriamine pentaacetic acid
ROC	Receiver operating characteristic
CT	Computed tomography
MRI	Magnetic resonance imaging
AFP	Alpha-fetoprotein
mRNA	Messenger ribonucleic acid
TCGA-LIHC	The Cancer Genome Atlas Liver Hepatocellular Carcinoma
OS	Overall survival
TCIA	The Cancer Imaging Archive
FPKM	Fragments Per Kilobase of transcript per Million mapped reads
TPM	Transcripts per million reads
ImmuCellAI	Immune Cell Abundance Identifier
GO	Gene Ontology
BP	Biological process
MF	Molecular function
CC	Cellular component
KEGG	Kyoto Encyclopedia of Genes and Genomes
ROI	Regions of interest
DEGs	differentially expressed genes
DICOM	Digital Medical Imaging and Communication
A.K	Artificial Intelligence Kit
mRMR	Maximum relative minimum redundancy
AIC	Akaike information criterion
ICCs	Interclass correlation coefficients
RS	Radiomics Score
ACC	Accuracy
SPE	Specificity
SEN	Sensitivity
PPV	Positive predictive value
NPV	Negative predictive value
AUC	Area under the curve
PR	Precision-recall
DCA	Decision curve analysis
CIs	Confidence intervals
HR	Hazard ratio.
